# Perceived organizational justice and turnover intention among hospital healthcare workers

**DOI:** 10.1186/s40359-020-0387-8

**Published:** 2020-02-22

**Authors:** Missaye Mulatie Mengstie

**Affiliations:** Department of Psychology, University of Gondar, P.O.Box:196, Gondar, Ethiopia

**Keywords:** Healthcare workers, Organizational justice, And turnover intention

## Abstract

**Background:**

Organizational justice is the first virtue in social institutions (J Manage 16:399-432, 1990). It is one of the most determinant factors for an effective utilization of human resources and an essential predictor of organizational success (J Manag Dev 28:457-477, 2009). Employees who perceive fairness are more likely happy with their job and less likely leave their organization (Int J Bus Manage 4:145-154, 2009). Perceived injustice, on the other hand, diminishes motivation of workers to accomplish their duties (Int J Bus Manage 4:145-154, 2009; J Educ Sci Univ Tabriz 2:27-34, 2009). Ethiopia has given emphasis to the expansion of health institutions and increasing the number of health professionals. Despite this, little emphasis has been given the human resource aspect of the health sector. Therefore, this study aims to investigate organizational justice perceptions and turnover intentions among healthcare workers in Amhara region.

**Methods:**

One hundred ninety seven healthcare workers participated in the study. Data were collected through self- report questionnaire and semi-structured interview. The quantitative data were analyzed through MANOVA, multiple regression, and independent samples t-test. The qualitative data were analyzed through thematic analysis.

**Results:**

The results of this study revealed that healthcare workers in the public hospitals held low perceived distributive, procedural, interpersonal and informational justice. Similarly, private hospitals healthcare workers had low perceptions on distributive and procedural justice. On the contrary, healthcare workers in private hospitals reported high perception of fairness on interpersonal and informational justice aspects. Both public and private hospital healthcare workers had high turnover intention. The result revealed significant difference in organizational justice perceptions between private and public hospital healthcare workers (F (4, 182) = 9.17; *p* < .05; partial η2 =. 168). Organizational justice dimensions (distributive, procedural, interpersonal and informational justice) significantly contributed an additional 9.9% variation in turnover intention (R ^2^ change = .099, F (4,170) = 4.86, *p* < .05). Distributive justice was the most important predictor of turnover intention (β = −.23, *p* < .05).

**Conclusion:**

Organizational justice perceptions of healthcare workers significantly predicted turnover intention. Hence, organizational justice should be given due emphasis in designing and implementing policies and strategies of human resource management.

## Background

Justice has a long history which attracted the interest of ancient philosophers like Plato and Socrates [1]. Though the inception of justice dates back to ancient years, the concept of organizational justice is a recent phenomenon. Scholarly interest in the area of organizational justice started in the 1970s [2]. Since then, organizational justice has received attention from various disciplines like Management and Social Psychology [3].

Organizational justice is a multidimensional construct which deals with everything from payment to treatment by one’s supervisor [1]. It is a judgment made by an employee about fairness of outcome distribution, processes in allocating outcomes and interpersonal relationships at the workplace (Greenberg, 1990). However, many of the organizational justice research were conducted in the USA, with particular emphasis to the business area [3]. Quite recently, scholars [4–6] have given considerable attention to workplace fairness in European and Asian countries.

Organizational justice has four dimensions including distributive, procedural, interpersonal and informational justice [1]. Distributive justice is concerned with fairness of outcomes like pay and promotion [7, 8]. It exists when there is a fair distribution of outcomes based on employees’ skills and contributions [9]. Employees perceive fairness of distribution of an outcome by comparing their own input-output ratio with that of others’ input- output ratio [2] Procedural justice concerns with the extent of fairness of procedures which are practiced in allocating outcomes to employees [2, 7]. It is all about fairness of processes that are used to decide how outcomes are distributed and to whom the outcomes are offered [2]. Interpersonal justice is the degree to which authority figures treat subordinates in respectful manner [2]. Informational justice concerns with the amount, authenticity and clarity of information regarding outcome distributions and the procedures used to determine outcomes [2, 7].

Organizational justice is the first virtue in social institutions [2]. People are concerned to issues of justice [10] and the question of workplace fairness is virtually the interest of employees regardless of size and setting of organizations [11]. Perceived injustice often diminishes motivation of workers to accomplish their duties [12, 13]. A large number of studies [7, 14–19] revealed that organizational justice is a consistent and significant predictor of employees’ organizational commitment, job satisfaction and turnover intention across various settings.

Effectiveness of an organization largely depends on its human and non-human resources [12]. Qualified human resources help an organization to succeed and survive in the competitive global market [10, 20]. However, qualified human resource in itself may not warrant productivity with little or no practice of justice at the workplace [12].

Ethiopia has made remarkable progress in health facility constructions and health professionals training [21]. In spite of this achievement, the overall proportion of health workers to the total population was only 2.30 per 10,000 [22]. More specifically, the proportion of health professionals to the total population was estimated to be 0.30 physicians, 2.30 clinical nurses and 0.20 midwifes per 10, 000 people [23]. Coming to the Amhara region, the ratio of physicians, health officers, nurses, midwives and health extension workers to the total population was estimated to be 1:58,567, 1:41,024, 1:4698 1:83,983 and 1:2383, respectively [22]. These show that Ethiopia did not meet World Health Organization’s minimum key indicators of health interventions which require 80% coverage of birth deliveries by skilled health attendants [24]. Covering 80% of birth deliveries by skilled health attendants requires 2.30 physicians, nurses and midwives per 10,000 people [24]. Thus, Ethiopia was listed among the 57 countries with a serious shortage of health workers. This was stated with the fact that it could not fulfill the health threshold of 80% coverage rate for deliveries by health attendants [24].

The shortage of medical doctors appears to be the most serious problem in the health sector of Ethiopia. The proportion of medical doctors to the total population in Ethiopia was 0.3 per 10, 000 people [25]. This is not only a concern in Ethiopia but countries around the world are also experiencing a shortage of healthcare workers [26]. Turnover of healthcare workers has been mentioned as one of the major factors for shortage of healthcare workers, especially medical doctors. One time it was reported that more Ethiopian doctors were working in the city of Chicago than in Ethiopia [27]. The number of Ethiopian doctors who migrated to foreign countries within a year was greater than the number of new medical graduates during the same year [28]. The public health sector has lost a large number of its employees due to turnover [28]. For instance, between 1987 and 2006, 73% of medical doctors left the public health centers and more than 80% of the public hospitals faced a serious shortage of medical doctors [28].

The Amhara regional is the second largest region in Ethiopia with a total population size of 20,558,851 [29]. The region was one of the most affected regions in the turnover of physicians. Taking into account its population size, the Amhara region had the lowest number of physicians [28]. There were 133 physicians in the region in 2005 and number drastically dropped to 68 in 2006 [28]. A survey study in the public health centers at Bahir Dar unveiled that the attrition rate of health professionals is 39.6% [30]. In addition, a study conducted among nurses in referral hospitals of the Amhara regional indicated that 60.2% of participants reported intention to leave their job [31]. Another survey among health professionals in the University of Gondar Referral Hospital indicated that more than half of the respondents (52.2%) reported turnover intention [32]. Another survey study among nurses in governmental health institutions of East Gojjam indicated that more than half of the respondents (59.4%) had turnover intention [33]. These imply high turnover intention among healthcare workers in the Amhara region. Eventually, losing a large number of healthcare workers at national and regional levels may result in a fragile health system that hampers delivery of services.

Many studies in Ethiopia revealed high turnover of health professionals [28, 34, 35]. The problem was the worst in public hospitals located outside Addis Ababa [28]. For instance, 50% of nurses in the Sidama Zone expressed intention to leave their organization [36]. Nevertheless, the Health Policy, the Health Sector Development Program IV (2010/11–2014/15) and the Growth and Transformation Plan (GTP) have not given specific mechanisms to retain the health work force. The main focus of these documents is expansion of health institutions and increasing the number of health professionals, giving little emphasis to human resource. There is lack of interest to examine whether or not organizational justice perceptions influence turnover intentions of healthcare workers. To date, there is one published study that indicated the role of distributive and procedural justice in explaining vulnerability to brain-drain among employees in higher learning institutions of Ethiopia [37]. Hence, the current study aimed to answer the following research questions.

- What is the organizational justice perception of healthcare workers in public and private hospitals?
- What is the level of turnover intention among healthcare workers in public and private hospitals?
- Do organizational justice dimensions significantly predict turnover intention among public and private hospital healthcare workers?

## Methods

### Aim

This study aims to investigate perceived organizational justice and turnover intention among healthcare workers in public and private hospitals at Bahir-Dar and Gondar cities, Ethiopia.

### Design

This study follows a mixed research method with convergent parallel (QUAN + qual) design. The convergent parallel design combines qualitative and quantitative methods. **T**he quantitative-qualitative interaction has become the most useful and popular method to investigate a research problem in multiple ways [38].

### Setting

This study was conducted in public and private hospitals in Bahir- Dar and Gondar cities, Amhara Region. Public hospitals are owned by the government to deliver healthcare services with relatively lower prices. Private hospitals, on the other hand, are business enterprises established and owned by individuals to make profit. To date, there are four hospitals in Bahir Dar and Gondar cities. Felege-Hiwot Referral Hospital and Gondar University Referral Hospital are public hospitals whereas GAMBY Teaching General Hospital and IBEX General Hospital are privately owned hospitals. These hospitals were selected for three main reasons. First, it is easy to find heterogeneous health professionals. But, it is difficult to find all health professionals in other small healthcare centers like clinics and drug stores. Hence, a hospital setting is better in terms of exploring the views of different health professionals. Secondly, it was convenient to access these cities that reduce research expenses. Thirdly and most importantly, as mentioned earlier, the Amhara region is one of the most affected regions in turnover of health professionals which needs emprical evidence that inform policy and practice.

### Participants and sampling techniques

According to the Human Resource Departments of the hospitals, there were 740 healthcare workers in public hospitals (361 healthcare workers in Felege-Hiwot Referral Hospital and 379 in Gondar University Referral Hospital). There were also 158 healthcare workers in the private hospitals (98 in GAMBY Teaching General Hospital and 60 in IBEX General Hospital). Overall, the total number of healthcare workers in the four hospitals was 898.

Respondents of the quantitative data were selected through disproportionate stratified random sampling technique. This sampling technique was used for two main reasons. First, selecting participants through disproportionate stratified random sampling helps to represent healthcare workers from each hospital. Secondly, since private hospitals have a smaller number of health workers compared to public hospitals, disproportionally selecting participants enables to avoid under representation of the private hospitals.

Stratification of the target population (healthcare workers) was made by hospitals. After forming four stratum (stratum 1 = Felege-Hiwot Referral Hospital, stratum 2 = Gondar University Referral Hospital, stratum 3 = GAMBY Teaching General Hospital, and stratum 4 = IBEX General Hospital), representative sample of participants were randomly selected from each stratum. Most participants were accessed in the medical wards of the hospitals (large rooms where a mix of health professionals treat patients) while other participants (e.g., pharmacists and psychiatrists) were accessed in their private office.

Two hundred ten participants were selected from the total eight hundred ninety eight healthcare workers. One hundred forty five participants were randomly selected from public hospitals and the remaining sixty five participants were randomly taken from private hospitals. Nevertheless, seven participants did not to return the questionnaires and eight participants failed to complete all the items. Besides, the Cook’s Distance test revealed outlier scores for eight participants and hence were discarded from the analysis. Given this, the responses of 187 participants (127 participants from public hospitals and 60 participants from private hospitals) were used for the analysis. It needs to take a minimum of 105 samples to represent a population size of 900 [39]. Based on this calculation, a sample size of 187 participants is believed to be representative of the total 898 health staff. In addition, power analysis was conducted through G* power software to estimate the sample size. Using alpha = 0.05 and power = 0.80, a sample size of 187 participants was found to be adequate to compute the statistical techniques (one way MANOVA, multiple regression and independent samples t-test).

In the case of the qualitative data, participants were selected through purposive sampling technique. Ten informants were purposely selected from four hospitals. Of these, six interviewees (2 doctors, 2 nurses, 1 health officer and 1 pharmacist) were selected from public hospitals. The remaining four interviewees (1 nurse, 1 laboratory technician and 2 pharmacists) were selected from private hospitals. From the total ten interviewees, 6 of them were females and the remaining 4 were males.

The criteria for selecting interviewees were work experience and department were considered to select interviewees. It is believed that employees who have worked a longer time in would have detail information about their organization. Hence, healthcare workers who had at least 2 years of job experience in their respective hospitals were purposely selected for interview. Besides, interviewees were purposely selected from different departments so as to consider a variety of perceptions. But, medical doctors from the private hospitals were only willing to give their response for items in the self- reported questionnaire.

### Measures

All the quantitative data scales were adapted from the original sources. The instruments were translated from English to the local language (Amharic) by three Social Psychologists and one Clinical Psychologist. Again, the Amharic version was translated back into English by the researcher. The language equivalence of the Amharic and the English versions of the instrument were checked by English language expert.

The clarity, wording and ordering of the final items were also checked by the three Social Psychologists who were involved in translation. They agreed upon the appropriateness and clarity of most of the items in the scales. They also forwarded suggestions to modify the wordings of some items. Based on their suggestions, some items were re-phrased and incorporated into the final instruments (Additional file 1).

#### Organizational justice measure

Organizational justice perception of participants was measured by justice scale [40]. The items were rated on a scale ranging from 1(strongly disagree) to 5 (strongly agree). The justice scale consists of 20 items (4-item measure of distributive justice that focuses on employees’ perception of fairness in outcome distribution, 7- items procedural justice scale that measures fairness perception in the processes and methods used to determine outcome distributions, 4-items scale of interpersonal justice that measures the extent to which authority figures treat employees with respect and 5- item scale of informational justice that measures the extent to which adequate information are provided to employees. Sample items include “To what extent do your pay and rewards reflect the effort you have put into your work? To what extent have you been able to express your views and feelings during those procedures used to determine your pay and rewards? To what extent has your supervisor treated you in a polite manner? To what extent has your supervisor communicated details in a timely manner?”

#### Turnover intention measure

This scale measures voluntary readiness of healthcare workers to quit working in their organization [41]. Turnover intention of participants was measured by a 5-item turnover intention measure on a scale ranging from strongly disagree (1) to strongly agree (5). A sample item is “I am very interested in job announcements or job opportunities outside of this hospital.”

#### Semi- structured interview

**A** face -to- face interview was conducted with key informants. The interview sessions took between 45 min and 1 h. Interviews were conducted at the offices of the informants. During the interview, notes were taken and conversations were recorded. Sample items include *“*How do you evaluate the fairness of procedures (methods) to decide distribution of outcomes? Do you have intention to quit working in this hospital? If so why and in what type of organization do you plan to work?”

### Pilot study

A pilot study was conducted to check the reliability and validity of the instruments. To this end, 70 participants (20 from private and 50 from public hospitals) were randomly selected and their responses were used to analyze the reliability and construct validity of the quantitative data measures.

### Reliability of the instruments

The reliability of the sub-scales was checked by Cronbach alpha. Table 1 shows the original, pilot study and main study reliability coefficients of the instruments. Both the pilot and the main studies indicated acceptable Cronbach alpha coefficients.

**Table 1:** Cronbach alpha coefficients of the instruments

Constructs	Sub-scales	No.of items	Original Alpha	Pilot Alpha	Main study Alpha
Organizational Justice	Distributive justice	4	.89	.86	.86
	Procedural justice	7	.84	.84	.86
	Interpersonal justice	4	.92	.88	.90
	Informational justice	5	.93	.91	.92
Turnover Intention	Turnover intention	5	.89	.81	.77

#### Content validity

The content validity of the scales was examined by a group of panelists consisting of eight members. The panelists evaluated the essentiality of each item based on three options (essential, useful but not essential and not necessary). The calculated values indicated that the content validity ratio of all items was greater than the minimum value (.75).

#### Construct validity

This was conducted to check the construct validity of the instruments. The rotated matrics factor loadings indicated adequate construct validity for all the instruments.

### Data collection procedures

Initially, assistant data collectors were given brief orientation about the procedures of collecting the quantitative data. Participants were asked about their willingness to give response. They were assured of the confidentiality and anonymity of their responses. After assuring this, a brief orientation was given to the participants about the purpose the study. Then, the assistant data collectors and the researcher distributed questionnaires in the workplace (medical wards and private offices) of participants. In the case of the qualitative data, the researcher conducted all the interview sessions in their office.

### Data analysis techniques

Inferential and descriptive statistics were used to analyze the quantitative data. Predictions of the independent variables on the dependent variables were computed using multiple regression. Independent samples t-test was used to check if there is a significant difference in turnover intention between public and private healthcare workers. One way MANOVA was computed to check if a significant difference exists in organizational justice perceptions between public and private healthcare workers.

In the case of the qualitative data analysis, interviewees narrated an account of their experiences regarding organizational justice perceptions and turnover intention. The researcher took notes in parallel with recording conversations. The researcher translated the recorded Amharic conversation into English. The translated English versions were again transcribed into text. The transcripts and the written notes were analyzed through thematic analysis. In doing the analysis, the tape recorded interview data were listened repeatedly. After exhaustive reading of the transcripts, the researcher generated codes from the entire data. The coding processes were manually performed. After generating codes, the relevant data were organized under each code whereas the marginal data were discarded. This led to establishment of patterns. Eventually, themes were emerged by organizing similar issues into categories. The themes were described and narrated.

### Ethical considerations

First of all, a letter of permission was collected from the School of Psychology, Addis Ababa University. In order to confirm acceptance, the letters were submitted to the medical directors of each hospital. After receiving permission from the medical directors, participants were also asked their willingness to participate in the study. Besides, approval was obtained from the Ethical Clearance Review Committee of College of Social Sciences and the Humanities, University of Gondar. Besides, written informed consent was obtained from participants before the commencement of data collection.

## Results

### Demographic information

Table 2 shows demographic information of participants. Proportional number of male (51.18%) and female (48.82%) public hospital healthcare workers participated in the study. The average age of participants was 29 year with a standard deviation of 6 year. The Largest proportions of participants in the public (65.4%) and private (65%) hospitals were nurses. Coming to educational level, most participants (74.02%) in the public hospitals were a first degree holder whereas most participants (71.67%) in the private hospitals were diploma holders. With respect to work experience, the average was 5.8 years, with the minimum and maximum ranges between 1 and 34 years. Finally, the maximum and minimum monthly salary of healthcare workers in private hospitals were 1000 and 45, 000 Ethiopian birr, respectively. In public hospitals, the maximum salary was 8500 Ethiopian birr and the minimum was 1123 Ethiopian birr. This shows a very large disparity in the amount of monthly salary especially in private hospitals.

**Table 2:** Demographic characteristics of respondents

	Public Hospitals		Private Hospitals	
Demographic variables	N	%	N	%
Gender				
Male	65	51.18	24	40
Female	62	48.82	36	60
Total	127	100	60	100
Job Title				
Medical doctor	11	8.7	6	10
Nurse	83	65.4	39	65
Pharmacist	12	9.4	4	6.7
Lab. Technician	10	7.9	9	15
Health officer	6	4.7	1	1.7
Midwifery	1	.8	1	1.7
Psychiatrist	4	3.1	–	–
Total	127	100	60	100
Education Level				
Diploma	26	20.47	43	71.67
First degree	94	74.02	11	18.33
Second degree	7	5.51	6	10
Total	127	100	60	100
Age				
Maximum	21		56	
Minimum	46		22	
M	27.83		29.6	
SD	5.5		6.33	
Salary				
Maximum	45,000		8600	
Minimum	1000		1123	
M	4491.74		3691.1	
SD	10,396.6		1380.55	

### Organizational justice perception among participants

The mean and standard deviation scores of participants on organizational justice perception dimensions are presented in Table 3. The results demonstrated that participants from public hospitals had mean scores of 7.36 on distributive justice, 13.25 on procedural justice, 10.25 on interpersonal justice and 13.59 on informational justice. The mean scores indicated that participants working in a public hospital had low perception of fairness across all dimensions of organizational justice and all the results were statistically significant (*p* < .05).

**Table 3:** Organizational justice perceptions of participants in public and private hospitals

Organizational justice dimensions	Hospital type	M	SD	t	df	Sig	Test value
Distributive justice	Public	7.36	3.05	17.1	126	.000	12
	Private	8.35	3.39	8.32	59	.000	12
Procedural justice	Public	13.25	5.05	17.25	126	.000	21
	Private	17.33	5.78	4.91	59	.000	21
Interpersonal justice	Public	10.97	4.46	2.6	126	.011	12
	Private	13.60	3.31	3.74	59	.000	12
Informational justice	Public	13.59	5.42	2.93	126	.004	15
	Private	16.65	4.30	2.3	59	.004	15

Similarly, two interviewees (a nurse and a medical doctor) who were working in public hospitals emphasized on the absence of transparency and impartiality in the administrative procedures of the hospitals. For instance, an interviewee (medical doctor) from a public hospital (X2) said that the procedures used to make decisions are not clear and there is no room for employees to express their views and objections. He reported “when we ask about criteria and procedures on certain decision making processes, they [the administrators] associate our question to politics.” By the same token, the other interviewee (a nurse) from the same hospital described problems related to administrative procedures of the hospital. She said:

There are a lot of problems in the administration of public healthcare centers. Before joining this hospital, I worked in another public clinic for some years. I left the clinic due to problems related to the administration. I joined this hospital in search of a better working environment. But I have faced similar obstacles in this hospital. I have not seen better administration in this hospital*.*

An interviewee (a Health Officer) who was working in another public hospital (X1) expressed similar and more specific views with respect to procedural justice as follows:

There is bias in treating staff. Those workers who are party members [the present ruling party of the country] are favored. Most of the time, decisions are based on their [the administrators] personal feelings and interests. Besides, they [the administrators] only take care of those who have high academic qualification [doctors]. Because, they [administrators] think that the rest of the staff will go nowhere. Hence, they do not give attention to other staff members*.*

In support of the above interviewee (Health officer) a medical doctor from public hospital X1 reflected similar view on procedural justice. He stated that as far as the medical staff is concerned, he did not observe serious problems in implementing procedures. Of course, he disclosed the presence of some problems; but the problems were not much discouraging to the medical staff.

With respect to distributive justice, all interviewees in the public hospitals expressed that the inputs they invest are not comparable to the outcomes they receive. They said that their outcomes (pay and other benefits) are very low, considering their educational level, efforts, abilities and the time they invest. All of them agreed that they do not receive a deserved outcome.

Regarding informational justice, all interviewees from public hospitals reflected the same idea. They said that the information released from higher officials is not clear, adequate and timely. For instance, an interviewee (a nurse) from public hospital (X2) expressed her observation as follows:

Sometimes, messages from the administration office do not have the full information. At times, new information does not reach all employees. An employee may miss an opportunity for further education or training due to delay of information. Surprisingly, there are times we access information from somebody else outside the hospital*.*

With respect to interpersonal justice, a health officer from public hospital (X1) and a nurse from public hospital (X2) seriously emphasized the disrespecting manner of supervisors in treating employees. Though the remaining four interviewees in public hospital X1 & X2 had objections on the partiality of staff treatment by supervisors, they did not consider it as a very severe problem.

Coming to private hospitals, participants scored mean values of 8.35, 17.33, 13.60 and 16.65 on distributive, procedural, interpersonal and informational justice, respectively (See Table 3). These mean values indicated low perception of fairness on distributive and procedural justice whereas high perceptions of fairness on interpersonal and informational justice. The results were statistically significant (*p* < .05).

The qualitative and quantitative data yield similar results. All private hospital interviewees agreed that the outcomes they receive are not comparable to the inputs they invest (time, experience and expertise). All of the interviewees from private hospitals agreed that they do not have voice to influence decisions made by supervisors though they witnessed the presence of clear, unbiased and consistent procedures. Concerning informational justice, all of the interviewees from private hospitals witnessed that top officials passed clear and adequate information to employees. For instance, one interviewee (a pharmacist) from private hospital (Y2) said:

Clear and adequate information have been given from administrators via different ways of communication like letters, posters and e-mail. In case of vague information there is a room to ask clarification. Some employees may access information after some days. It is the responsibility of each employee to see information from the hospital’s website.

Finally, all interviewees from private hospitals appreciated the way they are treated by their supervisors and stated that their interaction with supervisors was friendly and smooth.

### Gender difference in organizational justice and turnover intention

As displayed in Table 4, there was no significant difference between male and female participants in distributive justice (t = 1.17, *p* > .05**),** procedural justice(t = .47, *p* > .05, interpersonal justice(t = .79, *p* > .05**)** and informational justice(t = .17, *p* > .05**).** Similarly, there was no significant difference in turnover intention(t = .65, *p* > .05) between male and female participants.

**Table 4:** Gender difference in organizational justice and turnover intention

Organizational justice dimensions	Hospital type	M	SD	t	df	Sig
Distributive justice	Male	89	7.96	1.172	185	.243
	Female	98	7.41			
Procedural justice	Male	89	14.76	.469	185	.640
	Female	98	14.37			
Interpersonal justice	Male	89	12.07	.789	185	.431
	Female	98	11.58			
Informational justice	Male	89	14.64	.168	185	.867
	Female	98	14.51			
Turnover intention	Male	89	16.1573	.650	185	.517
	Female	98	16.6122			

### Comparing organizational justice perceptions of public and private hospital participants

A one-way MANOVA result in Table 5 revealed a significant difference in organizational justice perceptions between participants working in public and private hospitals (F (4, 182) = 9.17; *p* < .05; partial η^2^ =. 168). The multivariate effect size was 0.168 which implies that 16.8% of variance in organizational justice perceptions was accounted for by hospital type.

**Table 5:** One-way MANOVA comparing organizational justice perceptions of public and private hospital participants

Variable	Pillia’s Trace	F	Hyp.df	Error.df	Sig.	Partial η^2^
Organizational justice dimensions	17	9.17	4	182	000	168

A test of between subjects’ effects was further examined and presented in Table 6. Considering Bonferroni adjusted alpha value of .01, there were significant differences between public and private hospital participants on procedural justice (F (1, 185) = 24.15; *p* < .01), interpersonal justice (F (1, 185) =16.44; *p* < .01) and informational justice(F (1, 185) = 16.69; *p* < .01). However, public and private hospital participants did not significantly differ in perceived distributive justice (F (1, 185) = 3.95; *p* > .01). The mean scores in Table 3 demonstrated that private hospital personnel held significantly higher perception of procedural justice (M = 17.33, SD = 5.78) than public hospital participants (M = 13.25, SD =5.05). Moreover, private hospital participants had significantly higher perception of fairness on interpersonal justice (M = 13.60, SD = 3.31) vis -a-vis public hospital participants (M = 10.97, SD = 4.46). Finally, participants working in private hospitals reported significantly higher perception of fairness on informational justice (M = 16.65, SD = 4.30) compared to those who were working in public hospitals (M = 13.59, SD = 5.42).

**Table 6:** Between subjects effects test comparing organizational justice perceptions of participants

Organizational justice dimensions	df	Error df	F	Sig.	partial η^2^
Distributive justice	1	185	3.95	048	021
Procedural justice	1	185	24.15	000	115
Interpersonal justice	1	185	16.44	000	082
Informational justice	1	185	16.69	000	074

### Turnover intention among participants

Table 7 shows that both public hospital participants (M = 16.66, SD = 4.71) and private hospital participants (M = 15.81, SD = 4.89) had high turnover intention and did not significantly differ (t (185) = 1.25, *p* > .05). The magnitude of the difference in the means was also very small (partial η^2^ = .008). Though the difference was not significant and large, the turnover intention score of public hospital participants (M = 16.66) was slightly higher than private hospital participants (M = 15.88).

**Table 7:** Independent samples t-test comparing turnover intention between public and private hospital participants

Groups	Mean	SD	df	t	Sig.	Partial η^2^
Public hospital healthcare workers	16.66	4.71	185	1.25	26	008
Private hospital healthcare workers	15.81	4.89				

In support of the quantitative results, the qualitative results revealed that nine out of ten interviewees had intention to quit working in the hospitals. For instance, one interviewee (a medical doctor) in a public hospital (X1) stated:

Most medical doctors work until they finish their duty [obligation period of healthcare workers who graduated from a government university]. As a human being, I seek good opportunities. If I get the opportunity, I will leave this hospital. For instance, our medical director went to America to participate in a conference and he did not come back to Ethiopia.

Similarly another interviewee (a medical doctor in a public hospital X2) described his intention as follows:

My relatives have advised me to go abroad for better opportunities. But I resisted the pressure and became committed to serve society. At present, I am tired of issues related to the administration of the hospital. As a result, I have changed my mind and start thinking to leave working here [public hospital X 2]. There is a high turnover of doctors in this hospital. Though, this hospital was established earlier, it could not have attracted and maintained health professionals. Even, newly established hospitals are better in treating the medical staff than this hospital. Indeed, no one in the hospital stays if he or she finds alternatives. Some workers are counting down termination of their duty period*.*

Coming to private hospitals, three out of the four interviewees reported their plan to look for vacant post in other organizations. For instance, one interviewee (a pharmacist) in a private hospital (Y1) stated that she will never hesitate to quit working in the employing hospital if she finds a job in other organizations. She says “I work in this hospital so as to acquire better work experiences that will help me to join a better organization.” Overall, almost all interviewees in both private and public hospitals revealed turnover intention.

### Predicting participants’ turnover intention

Table 8 shows that demographic variables did not significantly explain turnover intention (R ^2^ = .036, F (4,174) = 1.61, *p* > .05). Taken together, demographic variables and organizational justice dimensions in Model 2 significantly predicted 13.5% of the variance in turnover intention (R ^2^ = .135, F (8,170) = 3.31, *p* < .05). After controlling the possible effects of demographic variables on turnover intention, organizational justice dimensions significantly contributed additional 9.9% variation in turnover intention (R ^2^ change = .099, F (4,170) = 4.86, *p* < .05). Among the four organizational justice dimensions, distributive justice was found to be the strongest predictor of turnover intention (β = −.23, *p* < .05).

**Table 8:** Organizational justice perceptions predicting turnover intention

Variables	Model 1 (β)	Model 2 (β)
Gender	.033	.037
Age	−.294*	−.18
Educational Level	−.032	.015
Experience	.246	.136
Distributive justice		−.23*
Procedural justice		−.074
Interpersonal justice		−.077
Informational justice		−.006
R ^2^	.036	.135
F for R ^2^	1.61	3.31**
R ^2^ change		.099
F for R ^2^ change		4.86**

Note: * = *p* < .05, ** = *p* < .01,

## Discussion

The present study revealed low perceived fairness of distributive and procedural justice among public and private hospital healthcare workers. This shows that the outcomes they receive from their employing hospital were inadequate as compared to their experiences, expertise and the amount of work they accomplish. This implies that the outcomes they received are not appropriate and do not reflect their contributions and performances. Similarly, [42] explained that when individuals’ outputs are lower than inputs, they feel distributive injustice and a fair distribution of outcomes leads to existence of distributive justice [9].

The present study indicated that organizational justice perception and turnover intention did not significantly differ by sex of participants. On the contrary, a study conducted by [43] demonstrated that females scored significantly higher on distributive, procedural and interactional justice perception than males.

The results of the present study indicated that decision making processes favored some employees, implying bias and inconsistency over individuals. The control model of procedural justice states that employees find opportunities to present their voice as indirect means of influencing decision- making processes [44]. If they are not allowed to have a voice and express their views, they are more likely to perceive procedural injustice [44].

The mean scores indicated high level of turnover intention among public and private hospital healthcare workers and organizational justice dimensions significantly contributed to turnover intention. Among the four organizational justice dimensions, distributive justice was the most important predictor of turnover intention. Similarly, other studies [45, 46] revealed that distributive justice has substantial unique variance associated to turnover intention than procedural, information and interpersonal justice dimensions.

## Conclusions

Both public and private hospital healthcare workers in Gondar and Bahir-Dar cities had low perception of distributive justice. The outcomes (pay and other benefits) they received from their employing hospitals were not fair compared to the inputs (experiences, expertise and the amount of work). The procedures and methods of decision-making in public were not also clear, consistent and free from bias. In addition, healthcare workers had no voice on the procedures of the hospitals and were not able to influence decision-making processes. Overall, the practices of distributive and procedural justice in both public and private hospitals were poor. Besides, healthcare workers in public hospitals did not receive clear and adequate information from supervisors. Besides, supervisors in public hospitals did not treat healthcare workers in respectful manner. Unlike public hospitals, supervisors in private hospitals treated employees with respect. Hence, private hospitals were performing better than public hospitals in practicing interpersonal and informational justice. Both public and private hospital healthcare workers had high turnover intention. Distributive justice (unfair distribution of outcomes) was the most determinant factor for high turnover intention of healthcare workers in the hospitals.

## Recommendation

The present study shows that most aspects of organizational justice were lacking in the hospitals, especially in the public ones. On the other hand, turnover intention of healthcare workers was high in both public and private hospitals. Hence, policy makers should also consider organizational justice perceptions and turnover intention of healthcare workers in planning health and health related policies. It is high time and paramount important for Federal Ministry of Health to give due emphasis to maintain the health professionals. This requires creating conducive working conditions (fairness or justice at workplace) and planning affordable salary and promotion opportunities.

## Supplementary information

**Additional file 1.** Questionnaire used to collect quantitative data. Interview guide used to collect qualitative data.

## Data Availability

The datasets analyzed during the current study are available in the Open Science Framework, https://osf.io/tpz4k/.

## Abbreviations

df: Degrees of freedom
M: Mean
MANOVA: Multivariate analysis of variance
SD: Standard deviation
