# Supplementary material for: Perceived organizational justice and turnover intention among hospital healthcare workers
Source: BMC Psychol. 2020 Feb 22;8:19. doi: 10.1186/s40359-020-0387-8 (PMC7036232; doi:10.1186/s40359-020-0387-8)
Supplement: Supplementary file 1 — Additional file 1. Questionnaire used to collect quantitative data. Interview guide used to collect qualitative data. [file 40359_2020_387_MOESM1_ESM.docx]

**Questionnaire**

**Objective**: The objective of this questionnaire is to assess organizational justice perceptions and turnover intentions of health care workers in private and public hospitals. Your response will be used for academic purposes and will be kept confidential. You are kindly requested to give your genuine responses to each of the questions. Thank you in advance for your cooperation.

**General background information**

In this section, you are kindly requested to give genuine response about your background information.

1. Gender: ___________

2. Age:____________

3. Educational level:_____________

4. Specialization:_____________

5. Job title: ___________________

6. Work experience: ___________

7. Monthly pay: _____________

**Organizational justice perception scale**

This section measures your perception about organizational justice in the hospital you are working. You are kindly requested to give your response to each question based on the following scale. Encircle the number that you choose.

1 = Not at all, 2 = Small extent, 3= Moderate extent, 4 = Great extent, 5 = Very great extent

| **S.N** | **Items** | Not at all | Small extent | Moderate extent | Great extent | Very great extent |
| --- | --- | --- | --- | --- | --- | --- |
| 1 | To what extent do your pay and rewards reflect the effort you have put into your work? | 1 | 2 | 3 | 4 | 5 |
| 2 | To what extent are your pay and rewards appropriate for the work you have completed? | 1 | 2 | 3 | 4 | 5 |
| 3 | To what extent do your pay and rewards reflect what you have contributed? | 1 | 2 | 3 | 4 | 5 |
| 4 | To what extent are your pay and rewards justified, given your performance? | 1 | 2 | 3 | 4 | 5 |
| 5 | To what extent have you been able to express your views and feelings during those procedures used to determine your pay and rewards? | 1 | 2 | 3 | 4 | 5 |
| 6 | To what extent have you had influence over the pay and rewards arrived at by procedures used to determine your pay and rewards? | 1 | 2 | 3 | 4 | 5 |
| 7 | To what extent have procedures been used to determine your pay and rewards been applied consistently? | 1 | 2 | 3 | 4 | 5 |
| 8 | To what extent have those procedures used to determine your pay and rewards been free of bias? | 1 | 2 | 3 | 4 | 5 |
| 9 | To what extent have those procedures used to determine your pay and rewards been based on accurate information? | 1 | 2 | 3 | 4 | 5 |
| 10 | To what extent have you been able to appeal the outcome arrived at by those procedures used to determine your pay and rewards? | 1 | 2 | 3 | 4 | 5 |
| 11 | To what extent those procedures used to determine your pay and rewards uphold ethical and moral standards? | 1 | 2 | 3 | 4 | 5 |
| 12 | To what extent has your supervisor treated you in a polite manner? | 1 | 2 | 3 | 4 | 5 |
| 13 | To what extent has your supervisor treated you with dignity? | 1 | 2 | 3 | 4 | 5 |
| 14 | To what extent has your supervisor treated you with respect? | 1 | 2 | 3 | 4 | 5 |
| 15 | To what extent has your supervisor refrained from improper or undesirable remarks or comments? | 1 | 2 | 3 | 4 | 5 |
| 16 | To what extent has your supervisor been candid in his/her communications with you? | 1 | 2 | 3 | 4 | 5 |
| 17 | To what extent has your supervisor communicated details in a timely manner? | 1 | 2 | 3 | 4 | 5 |
| 18 | To what extent has your supervisor seem to tailor communications to your specific needs? | 1 | 2 | 3 | 4 | 5 |
| 19 | To what extent has your supervisor explained the procedures thoroughly? | 1 | 2 | 3 | 4 | 5 |
| 20 | To what extent has your supervisor’s explanations regarding the procedures were reasonable? | 1 | 2 | 3 | 4 | 5 |

**Turnover intention scale**

The following items measure whether you have intention to quit your current job in the hospital you are working. You are kindly requested to give your response based on the following scale.

Strongly disagree = 1, Disagree = 2, Neutral = 3, Agree = 4, Strongly agree = 5.

| **SN** | **Items** | Strongly disagree | Disagree | Neutral | Agree | Strongly agree |
| --- | --- | --- | --- | --- | --- | --- |
| 1 | I am very interested in job announcements or job opportunities outside of this hospital. | 1 | 2 | 3 | 4 | 5 |
| 2 | I am looking actively for a position outside of this hospital. | 1 | 2 | 3 | 4 | 5 |
| 3 | I think it is not helpful for my career to work at this hospital. | 1 | 2 | 3 | 4 | 5 |
| 4 | I think it would be better to quit this hospital and move to another job. | 1 | 2 | 3 | 4 | 5 |
| 5 | If other conditions allow, I will quit this hospital immediately | 1 | 2 | 3 | 4 | 5 |

**Interview Guide**

**Objective**: The objective of this interview is to assess organizational justice perceptions and turnover intentions of health care workers in private and public hospitals. Your response will be used for academic purpose only and it will be kept confidential. Thank you in advance for your cooperation

**General Background Information**

1. Gender: ______________

2. Age: ______________

3. Educational level:______________

4. Specialization:______________

5. Job title:______________

6. Work experience:______________

7. Monthly payment:______________

**Semi- structured Interview Questions**

1. How do you describe fairness of reward (pay, promotion) distribution in your hospital?

Do you think that you get what you should deserve?

1. How do you evaluate the fairness of procedures (methods) to decide distribution of outcomes?
2. How do you see fairness interpersonal interaction between authority figures and subordinates?
3. Do authority figures properly dispense information to employees? If so how genuine, adequate and timely are the information?
4. Do you have the intention to quit working in this hospital? If so why and in what type of organizations do you plan to work?
